# The comparison of postoperative analgesic requirements between modified thoracoabdominal nerve block through perichondrial approach versus wound infiltration analgesia in patients undergoing gynecological laparoscopic surgery: a retrospective, exploratory study

**DOI:** 10.1186/s40981-023-00632-w

**Published:** 2023-06-24

**Authors:** China Atsumi, Katsuhiro Aikawa, Keita Takahashi, Kazufumi Okada, Yuji Morimoto

**Affiliations:** 1 Department of Anesthesiology and Critical Care Medicine, Faculty of Medicine and Graduate School of Medicine, Hokkaido University Hospital, Sapporo, 060-8638 Japan; 2 Department of Anesthesiology, Sunagawa City Medical Center, Sunagawa, Japan; 3 Promotion Unit, Data Science Center, Institute of Health Science Innovation for Medical Care, Hokkaido University Hospital, Sapporo, Japan

**Keywords:** Modified-thoracoabdominal nerve block through perichondrial approach (M-TAPA), Wound infiltration analgesia, Gynecologic laparoscopic surgery, Postoperative analgesia

## Abstract

**Introduction:**

Recently, modified thoracoabdominal nerve block through perichondrial approach (M-TAPA) has been introduced as a novel trunk block. To date, studies comparing its clinical advantages with those of existing local anesthetic techniques are scarce. We aimed to compare the analgesic efficacy of M-TAPA to that of wound infiltration analgesia (WIA) in patients who underwent gynecological laparoscopic surgeries.

**Methods:**

We studied medical records from January 2020 to July 2021 at Hokkaido University Hospital. The primary outcome was the number of analgesic requirements in the first 24 h postoperatively. Secondary outcomes were the time until the first analgesic requirement and adverse events regarding local anesthetic techniques. To address confounding, a regression model was used.

**Results:**

Data from 90 of 231 patients were analyzed (M-TAPA group, *n* = 40; WIA group, *n* = 50). For the primary outcome, means and 95% confidence intervals for each group and between-group differences were as follows: 2.25 (1.74, 2.76), 2.28 (1.81, 2.75), and −0.03 (−0.72, 0.66), respectively. Adjusted mean difference was 0.39 (−0.32, 1.11). There were no significant differences in means between groups, with or without adjustment for covariates (*p* = 0.93, 0.28). Furthermore, no significant difference was detected in the time until the first analgesic requirement and adverse events related to local anesthesia.

**Conclusion:**

Our results demonstrate that M-TAPA did not reduce postoperative analgesic requirements compared to WIA. In a future clinical trial, sufficient visceral pain control may be required to evaluate the effectiveness of M-TAPA over WIA in patients undergoing laparoscopic gynecological surgery.

## Background

Laparoscopic surgery for gynecological disease is widely performed due to the advantage in cosmesis, postoperative pain, and morbidity [1]. Although it is less invasive than open laparotomy, patients undergoing laparoscopic surgery often experience significant pain [2]. Uncontrolled acute postoperative pain is associated with impaired physical function and prolonged opioid use, which consequently retards early recovery [3]. To provide effective analgesia and minimize opioid use, multimodal analgesia with non-opioid analgesics, such as local anesthesia, is recommended [4]. Transversus abdominis plane block (TAPB) [5], rectus sheath block (RSB) [6], and wound infiltration anesthesia (WIA) [7] are commonly used in various abdominal surgeries as components of multimodal analgesia.

Recently, modified thoracoabdominal nerve block through perichondrial approach (M-TAPA) [8] has been introduced as a novel technique of trunk block. Some reports have demonstrated that M-TAPA could be beneficial for lower laparotomy [9], sleeve gastrectomy [10], and laparoscopic cholecystectomy [11, 12]. We previously reported that this technique could provide a broad range of analgesia in the thoracoabdominal wall, with significant inter-individual variation [13]. Although a cumulative number of successful cases has been reported, few studies have evaluated the clinical advantage of M-TAPA over existing local anesthetic techniques [12]. Some previous studies indicate that trunk block provides longer analgesia than WIA [14]. Thus, in this retrospective, exploratory study, we hypothesized that M-TAPA could reduce the postoperative analgesic requirements compared to WIA in patients undergoing gynecological laparoscopic surgery.

## Methods

### Patient selection

This single-center retrospective study was conducted in accordance with the Declaration of Helsinki and approved by the Ethical Review Committee of Hokkaido University Hospital (IRB No. 021-0077; Nov 26, 2021). We aimed to compare the analgesic effectiveness of M-TAPA to that of WIA in patients who underwent gynecological laparoscopic surgeries. Written informed consent was not obtained owing to the retrospective design of this study. Consent was obtained through an opt-out method, and the opportunity to refuse was provided through the Hokkaido University Hospital website (https://www.huhp.hokudai.ac.jp/date/rinsho-johokokai/approval/2021-11/).

We studied the medical records of patients who underwent gynecological laparoscopic surgery between January 2020 and July 2021 at Hokkaido University Hospital. Exclusion criteria were as follows: emergency surgery, age < 20 or ≥ 70 years, surgery duration ≥ 6 h, no use of local anesthetic, or provision of peripheral nerve blocks other than M-TAPA (i.e., TAPB, RSB). Notably, we excluded patients who received both flurbiprofen and acetaminophen or neither of them intraoperatively, as this could influence postoperative analgesic requirements.

As “gynecological laparoscopic surgery” includes different procedures, and this might influence the results; we classified the types of surgical procedures into four groups: myomectomy with or without ovarian surgery, total vaginal hysterectomy with or without ovarian surgery, cauterization of endometriosis, and ovarian surgery only, according to previous studies [15, 16].

### Management of general anesthesia

Anesthetic and perioperative management were performed according to our institutional practices. Of note, although we usually select total intravenous anesthesia (TIVA) using propofol in this population, the anesthetic method differed because of the unstable supply of propofol occasioned by the coronavirus disease pandemic during the study period. Preoperative sedatives or analgesics were not administered. In the operating room, all patients were monitored for noninvasive blood pressure, electrocardiography, and peripheral oxygen saturation. A venous line was secured prior to induction. The anesthetic method was determined by the anesthesiologist in charge. TIVA was maintained with propofol (2.5–4 μg/mL) and remifentanil (0.1–0.5 μg/kg/min), targeting a bispectral index (BIS) value of 40–60. Inhalational anesthesia was maintained with 0.7–1 minimum alveolar concentration of end-tidal sevoflurane or desflurane and remifentanil. Intraoperative fentanyl administration was determined by an assigned anesthesiologist. All patients were intubated after the administration of rocuronium (0.6 mg/kg) and maintained with end-tidal carbon dioxide at 35–45 mmHg. Additionally, either of acetaminophen or flurbiprofen was given for postoperative pain control.

### Surgical procedures and local anesthetic technique

In our institution, gynecological laparoscopic surgeries are performed through the 4-trocar procedure combining 12 mm-, 10 mm-, 5 mm-thick ports. The port sites are umbilicus, suprapubic midline, and right and left lower abdomen. Generally, 10-mm- or 12-mm-thick ports, mainly associated with postoperative somatic pain, are inserted in the umbilicus and right lower quadratus.

**Fig. 1:**
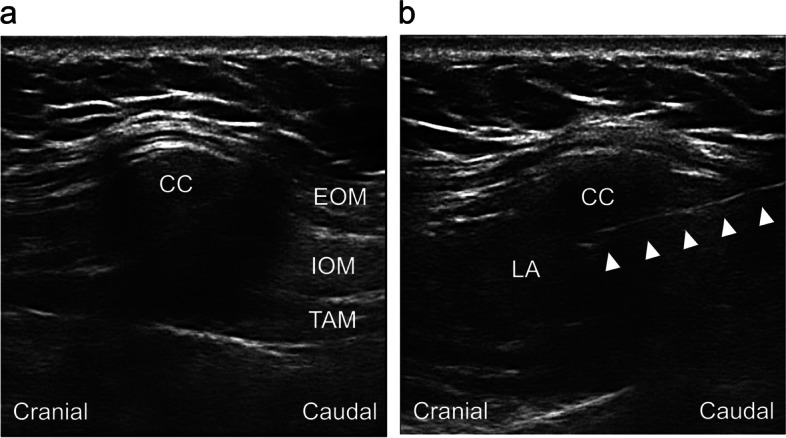
Ultrasound image of M-TAPA. **a** An appropriate image for M-TAPA. **b** Image obtained after injecting the local anesthetic solution. The arrows denote the Touhy needle. *M-TAPA* modified thoracoabdominal nerve block through perichondrial approach, *EOM* external oblique muscle, *IOM* internal oblique muscle, *TAM* transversus abdominis muscle, *CC* costal cartilage, *LA* local anesthetic

**Fig. 2:**
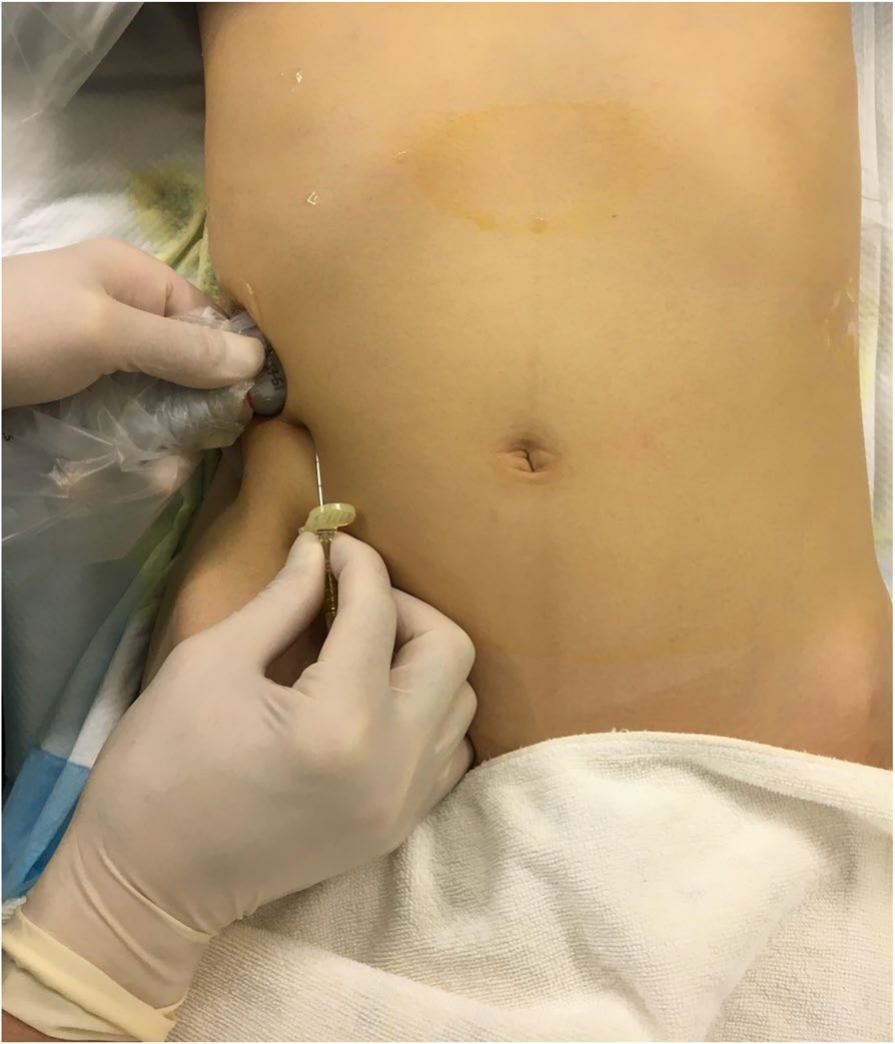
Ultrasound probe and needle position on the body surface during the performance of M-TAPA

M-TAPA was performed by a single anesthesiologist (KA) after induction of general anesthesia as previously described [8, 13]. Briefly, a high-frequency linear probe (EDGE, Sonosite, Tokyo, Japan) was positioned across the costal arch, and the four key structures were visualized: external oblique muscle, internal oblique muscle, transversus abdominis muscle, and costal cartilage. An 18-gauge Touhy needle was then inserted from the caudal to cranial direction using the in-plane technique until the needle tip was positioned between the posterior aspect of the costal cartilage and the transversus abdominis muscle. Twenty-five milliliters of 0.25% ropivacaine was injected on each side (Fig. 1a, b). A picture of the body surface during the procedure is shown as Fig. 2. We note that this group included 30 patients who were involved in our prior prospective study evaluating sensory coverage [13].

In the WIA group, local anesthesia was administered by the surgeons at the time of wound closure. There were differences in the types and volumes of local anesthetic solutions. However, in most cases, 10–20 mL of 0.25% levobupivacaine or 0.375% ropivacaine was injected around the port incision site.

### Postoperative pain management in the ward

After emergence from anesthesia and confirmation of a modified Aldrete score of ≥ 9, the patients were discharged to the gynecological ward. As the ward nurses were not informed of which local anesthetic technique was utilized, this study was considered to be observer-blind. In our institution, to avoid postoperative nausea and vomiting, intravenous opioid infusion was not used for patients who underwent laparoscopic gynecological surgery. Instead, intravenous acetaminophen, flurbiprofen, or intramuscular pentazocine (relatively rare) was administered at the patient’s request. Usually, the nurses administered acetaminophen or flurbiprofen first and then chose either analgesic to ensure an interval of at least 6 h between the same drugs. In addition to these rescue analgesics, 60 mg of scheduled oral loxoprofen was initiated from the next day of operation.

### Outcome measures and statistical analysis

The primary outcome was the number of postoperative analgesic requirements other than the scheduled oral loxoprofen in the first 24 h. Secondary outcomes were the time until the first analgesic requirement and adverse events associated with the local anesthetic technique in the perioperative period.

Due to the retrospective design of this study, the sample size was determined based on the number of patients enrolled during the study period. Assuming that M-TAPA reduced the postoperative analgesic requirements by 1 compared to WIA with a standard deviation of 2, with a two-sided significance level of 0.05%, the target sample size was 50 patients per group for a power of 70%. During the study period, more than 200 gynecological laparoscopic surgeries were expected to be performed. Even if 50% of the patients met the exclusion criteria, we anticipated to have a sufficient sample size to assess the difference in postoperative analgesic requirements between M-TAPA and WIA. Patient characteristics are presented as mean ± standard deviation. For the primary outcome, means for each group and between-group differences were estimated.

To adjust for confounding, a regression model with types of surgical procedures, patient body mass index, administered intraoperative non-opioid analgesics (acetaminophen or flurbiprofen), and anesthetic method (TIVA or inhalational anesthesia) as covariates was used to estimate the least-squares mean for between-group differences. All the estimates are shown with their 95% confidence interval (CI). Two-sided *p*-values for the between-group comparison were also calculated. The time until the first analgesic requirement was compared using Kaplan–Meier method and log-rank test. All statistical calculations were performed using JMP Pro 16 software (SAS Institute, Japan). Data were compared between groups using a two-tailed Student’s *t*-test. The categorical data were analyzed using the chi-square test. A *p*-value of < 0.05 was considered significant.

## Results

### Eligibility for analysis

**Fig. 3:**
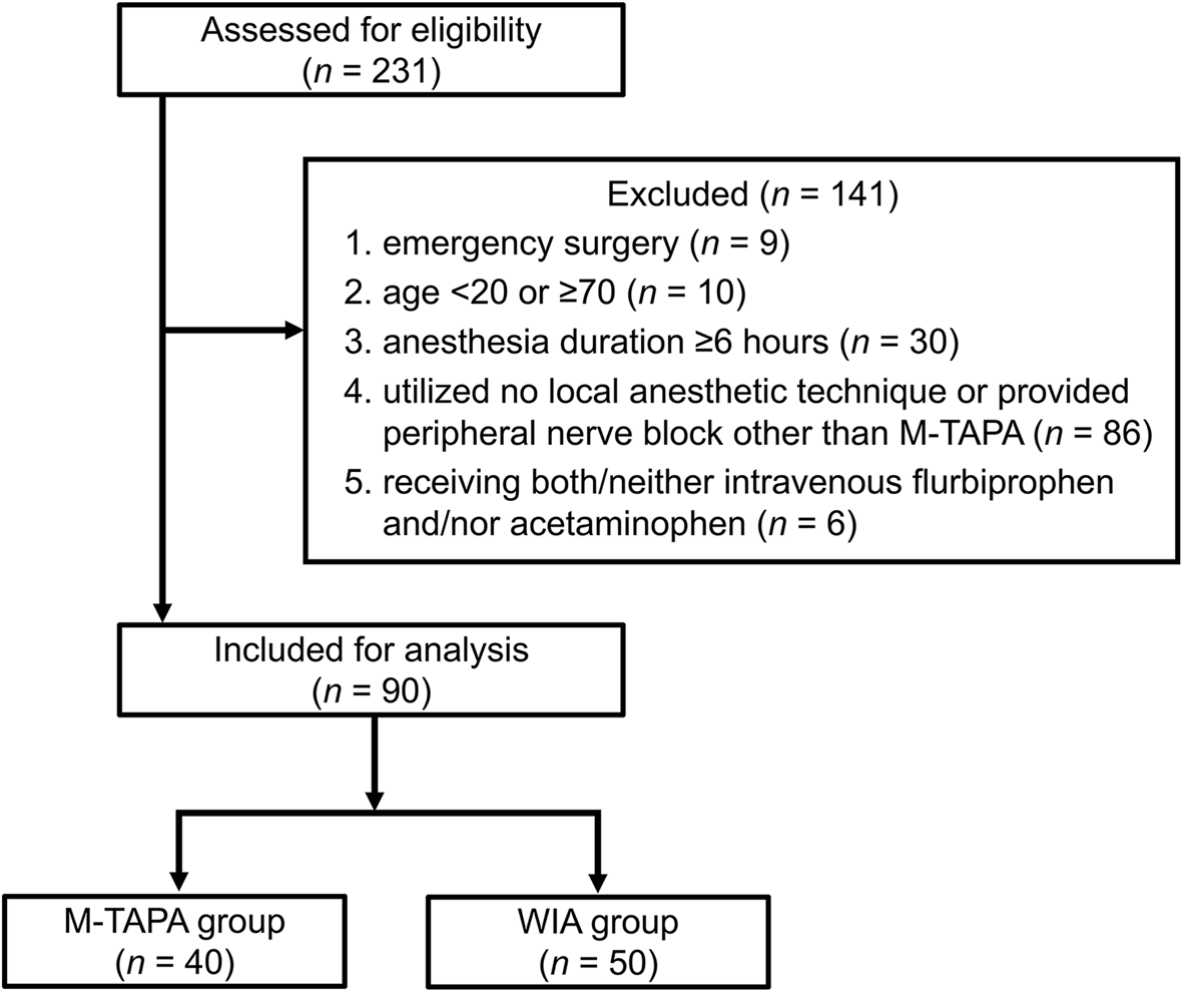
CONSORT flow diagram

A total of 231 gynecological laparoscopic surgeries were performed during the study period. We excluded the following: emergency surgery (*n* = 9), age < 20 or ≥ 70 years (*n* = 10), surgery duration ≥ 6 h (*n* = 30), use of no local anesthesia or peripheral nerve block other than M-TAPA (*n* = 86), and administration of both intravenous flurbiprofen and acetaminophen or neither of them (*n* = 6). Of the remaining 90 patients, 40 were in the M-TAPA group, and 50 were in the WIA group (Fig. 3).

### Patient characteristics and statistical analysis

Patient characteristics are listed in Table 1. There were no significant differences except for patient body weight, body mass index, and anesthetic method.

**Table 1:** Patient demographics

	M-TAPA group (*n* = 40)	WIA group (*n* = 50)
Age	42.9 ± 12.0	44.1 ± 11.2
Height (cm)	158.4 ± 4.9	158.1 ± 6.1
Weight (kg)	58.5 ± 12.6	65.2 ± 18.2
BMI	23.3±4.7	26.1±7.1
Anesthesia duration (min)	201.5 ± 69.0	222.2 ± 76.8
Intraoperative fentanyl (μg/kg)	4.5 ± 1.8	4.8 ± 2.4
Intraoperative remifentanil (μg/kg/min)	0.22 ± 0.05	0.23 ± 0.06
Intraoperative analgesics (acetaminophen/flurbiprofen)	15/24	20/23
ASA-PS (1/2/3)	18/21/1	17/27/6
Surgical procedures (M/T/C/O)	4/6/13/17	8/18/10/14
Anesthetic method (TIVA/inhalational)	36/4	34/16

Data are shown as the mean ± standard deviation or number of stated events. *M-TAPA* modified thoracoabdominal nerve block through the perichondrial approach, *WIA* wound infiltration analgesia, *BMI* body mass index, *ASA-PS* American Society of Anesthesia-Physical Status, *M* myomectomy with or without ovarian surgery, *T* total vaginal hysterectomy with or without ovarian surgery, *C* cauterization of endometriosis, *O* ovarian surgery only, *TIVA* total intravenous anesthesia

**Fig. 4:**
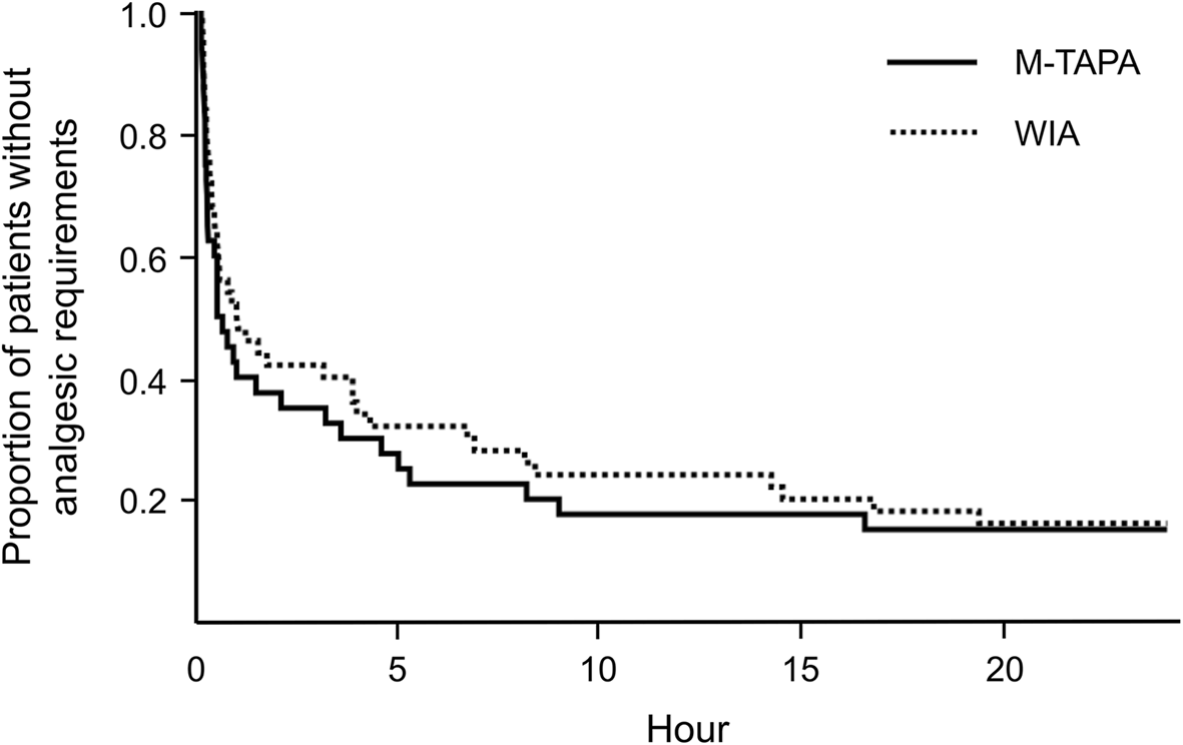
The Kaplan–Meier plot of the proportion of patients without analgesic requirement. The solid line represents M-TAPA group, and dotted line represents WIA group. *M-TAPA* modified thoracoabdominal nerve block through the perichondrial approach, *WIA* wound infiltration analgesia

Table 2 shows the number of postoperative analgesic requirements in the first 24 h. For the primary outcome, means and 95% CIs for each group and between-group differences were 2.25 (1.74, 2.76), 2.28 (1.81, 2.75), and −0.03 (−0.71, 0.65), respectively. The least-squares mean (with the regression model) for between-group difference was 0.39 (−0.32, 1.11). No significant differences were observed between groups, with or without adjustment for covariates (*p* = 0.93, 0.28). For the secondary outcomes, there was no significant difference in the time until the first analgesic requirement (*p* = 0.52, Fig. 4). Furthermore, no perioperative adverse events associated with local anesthetic techniques, such as toxicity or hematoma, were observed.

**Table 2:** Analgesic requirements in the first 24 h postoperatively

	Mean ± SD		Mean difference (95% *CI*)		*p*-value	
Analgesic requirements	M-TAPA group (*n* = 40)	WIA group (*n* = 50)	Unadjusted	Adjusted	Unadjusted	Adjusted
	2.25 ± 1.6	2.28 ± 1.65	−0.03 (−0.71, 0.65)	0.39 (−0.32, 1.11)	0.93	0.28

Data are shown as the mean ± standard deviation; mean (95% confidence interval). *SD* standard deviation, *CI* confidence interval, *M-TAPA* modified-thoracoabdominal nerve block through perichondrial approach, *WIA* wound infiltration analgesia

## Discussion

This retrospective study examined the analgesic efficacy of M-TAPA versus WIA in patients who underwent gynecological laparoscopic surgery by comparing the number of postoperative analgesic requirements and the time until the first analgesic requirement. The main finding of this study was that there was no difference in postoperative analgesic requirements between M-TAPA and WIA in the patients undergoing gynecological laparoscopic surgery. Furthermore, no difference in the time until the first analgesic requirement was observed.

M-TAPA is a novel trunk block, first reported by Tulgar et al. in 2019 [8]. To date, there is little research investigating its analgesic efficacy compared to existing local anesthetic techniques. Recently, Bilge et al. [11] reported the first randomized controlled trial indicating that M-TAPA decreased postoperative pain scores and tramadol consumption in the first 24 h and improved the quality of recovery-40 (QoR-40) scores after laparoscopic cholecystectomy compared with their standard postoperative analgesic regimen, without WIA. Subsequently, Güngör et al. [12] reported concordant results, utilizing WIA in the control group. These recent reports suggest that M-TAPA could be beneficial for laparoscopic cholecystectomy.

To the best of our knowledge, this is the first study investigating the analgesic efficiency of M-TAPA for laparoscopic gynecological surgery, which is often involved in studies regarding trunk peripheral nerve blocks. The discrepancy between the studies targeting laparoscopic cholecystectomy could be partially explained by the pain origin of the surgery performed. The postoperative pain after laparoscopic cholecystectomy mainly derives from the port incision site [17], which can be well controlled by M-TAPA. In contrast, pain after laparoscopic gynecological surgery mainly originates from visceral pain [18]. Kane et al. [16] reported that TAP block did not improve the postoperative quality of recovery-40 scores and pain scores or decrease postoperative opioid consumption in laparoscopic hysterectomy, which may support our results. Notably, half of the patients in both groups used the first rescue analgesic within 1 h, when the local anesthesia would be still effective [13]. Actually, they complained of “menstrual-like pain” at our postoperative rounds. Thus, our results were strongly influenced by visceral pain and suggest that sufficient visceral pain control is required in a future clinical trial to evaluate the effectiveness of M-TAPA over WIA in patients undergoing laparoscopic gynecological surgery.

When we evaluate the advantages of the novel nerve block technique, an adequate alternative local anesthetic technique should be provided to the control group to avoid overrating its clinical advantage [19]. Although some studies reported that trunk blocks provide longer analgesia than WIA [14], robust evidence that trunk blocks are clinically superior to WIA has not been shown. Of note, WIA has significant advantages over peripheral nerve blocks, as it does not require an anesthesiologist’s skill or ultrasound devices and is time-saving. Therefore, it is commonly used for multimodal analgesia, and we suppose it could be an appropriate control to evaluate the clinical efficacy of M-TAPA. Although we performed M-TAPA before surgery, postoperative application could be an option in a future study, as it will provide longer analgesia for the incision site [12].

In the present study, several types of surgical procedures were performed in accordance with the clinical settings. Hysterectomy has been suggested to induce more severe postoperative pain than other procedures, such as ovarian surgeries [18]. Therefore, some studies included only hysterectomy to ensure uniformity of patient characteristics [16, 20]. Thus, we utilized a regression model to address this confounding, and the mean difference of the analgesic requirement between M-TAPA vs WIA changed from −0.03 to 0.39. This may reflect that the WIA group included more patients who underwent total hysterectomy.

Theoretically, M-TAPA performed before incision ameliorates somatic pain and reduces intraoperative opioid use. However, no significant differences were observed between the two groups. As this was a retrospective study, there were no specific criteria regarding intraoperative opioid administration. The assigned anesthesiologists might use a sufficient dose of remifentanil throughout the operation to control adrenergic reactions due to pneumoperitoneum or visceral pain. To investigate the effect of intraoperative analgesic reduction, a prospective study with appropriate anesthetic protocol may be required.

Our study has some limitations. This was a single-center, retrospective study that did not unify the anesthetic protocol or postoperative analgesic regimen. The sample size of the M-TAPA group was 10 participants smaller than our expectation. Thus, we cannot rule out the possibility of under-powering to detect the difference in primary outcome. The choice of the local anesthetic technique was not random, which may have resulted in a selection bias of patient characteristics. Furthermore, our study lacked chronological postoperative pain assessment using a scale such as VAS or NRS, due to the unavailability of medical records. This also meant that the objective criteria to use rescue analgesia (i.e., *NRS* ≥ 4) were absent. We utilized a regression model including the types of surgical procedures as one of the covariates to adjust for confounding; however, future prospective studies on patients receiving the same surgical procedure are needed for a more appropriate comparison.

## Conclusions

Our study demonstrated no difference between M-TAPA and WIA in the postoperative analgesic requirements in the first 24 h and the time until the first analgesic requirement in patients who underwent gynecological laparoscopic surgery. A future clinical trial with sufficient visceral pain control may be required to evaluate the effectiveness of M-TAPA over WIA in patients undergoing laparoscopic gynecological surgery.

## Data Availability

The datasets are available from the corresponding author on reasonable request.

## Abbreviations

M-TAPA: Modified thoracoabdominal nerve block through perichondrial approach
WIA: Wound infiltration analgesia
TAPB: Transversus abdominis plane block
RSB: Rectus sheath block
TIVA: Total intravenous anesthesia
BIS: Bispectral index
CI: Confidence interval
